# Eugenics and Ethics

**Published:** 1914-08

**Authors:** 


### The Department of Eugenics, Mental and Social Hygiene

CONDUCTED BY
DR. MALONE DUGGAN
813-814 Gibbs Building, San Antonio, Texas
All communications for this department should be sent to DR. DUGGAN

### Eugenics and Ethics.

The highest aim of society is to better mankind; to lessen the burdens of life and increase efficiency and contentment. Not to progress is to degenerate; it is the law of nature to advance. Human betterment is obtained by definite fundamental principles. The innate qualities are developed through heredity (eugenics); cultural qualities through social control (sociology). Eugenics seeks to eradicate bad traits and build up good ones; to evolve a race that would be strong mentally, physically, and morally. Dr. Barker has said: "If we could know the unite characteristics which both parents possess, as well as those which they both lack, and also the unite characters in which they differ, and if we further knew for each characteristic, whether it was due to a presence or absence, we could go far towards predicting the result of a particular marriage." If this were possible, race culture would be simply a matter of breeding. But the laws of heredity are not sufficiently known to govern this factor yet. There are strict limitations on human breeding. There is an ethical equation, not common to stock, when man is concerned, which is as fundamental as physical laws. It is not alone a question of artificial selection, or the survival of the fittest, but the survival of the *ethically best,* and ethical qualities are subject to entirely different laws than those that govern heredity.

In heredity only unite qualities can be inherited, not general resemblances nor acquired characters. Besides, there have been but few unite characters analyzed. Even so, were it possible to breed all characters as you wanted, there would still remain the ethical principles to be considered. If all men were bred up to one standard of moral and physical efficiency, they would fit very poorly into their environment. Only the best, i. e., the strongest morally would survive. If all men were bred to be statesmen or doctors, there would be no one capable of tilling the soil or driving the wheels of industry. Each generation determines its own environment (social control), and the individual adapts himself according to his *ethical fitness.*

The surroundings in which a child is born and brought up influence his character even more than his inherited tendencies, i. e., in determining his life's action. These influences are called by Dr. Sumner the "mores" and must be regarded as fundamental factors in social control. This is the great ethical problem in social development and must be fostered not only for the immediate good that it does, but for the larger development it affords for the permanent innate betterment of the race.

*Social Reform.*—From the foregoing, we can clearly see in what relation eugenics stands to social reform. If moral and intellectual traits could be bred like unite characters, we would have a race that would live morally, courageously, and patriotically because of their innate nature to do so and not because of repression, forced by social control. But the prostitute, the habituate, and the weak-minded are such because of their *mores* groups. For that reason moral reform must continue to be a problem of ethics rather than eugenics.

*Duty of the State.*—Whatever lesson this viewpoint may teach, there are serious and compelling conditions which the State must consider. For example, there can be no question of heritable unite characters being present in idiocy, epilepsy, and feeble-mindedness, and their control can only be assured by strict regulation of procreation. Marriage is for the social welfare, and parenthood is a social privilege. Therefore, the State must assume the responsibility for the character of the offspring. In no other way could personal liberty and equal rights be guaranteed. When the bearing of children is likely to become a burden on society, that privilege must be withheld. We are only free moral agents so long as our acts do not interfere with the rights of others. How much more is this truth emphasized in bringing into the world defective children.

It is not alone by segregation or sterilization that the State can exercise this duty, but also by intelligent instruction on the responsibility of parenthood and the mechanical means of regulating procreation.

Thus eugenics and ethics work hand in hand in the one great aim of human betterment. Ethics, through social culture, improves the immediate condition of society and makes it possible for eugenics to improve the innate qualities of the race by creating the necessary mores for its further understanding.

